# Glycation of fibronectin inhibits VEGF‐induced angiogenesis by uncoupling VEGF receptor‐2‐c‐Src crosstalk

**DOI:** 10.1111/jcmm.15552

**Published:** 2020-07-01

**Authors:** Tangting Chen, Jinling Dong, Haiyan Zhou, Xin Deng, Rong Li, Ni Chen, Mao Luo, Yongjie Li, Jianbo Wu, Liqun Wang

**Affiliations:** ^1^ Key Laboratory of Medical Electrophysiology Ministry of Education and Medical Electrophysiological Key Laboratory of Sichuan Province Collaborative Innovation Center for Prevention and Treatment of Cardiovascular Disease of Sichuan Province Institute of Cardiovascular Research Southwest Medical University Luzhou China; ^2^ Drug Discovery Research Center Southwest Medical University Luzhou China; ^3^ Laboratory for Cardiovascular Pharmacology of Department of Pharmacology The School of Pharmacy Southwest Medical University Luzhou China

**Keywords:** advanced glycation end products, angiogenesis, c‐Src, fibronectin, receptor for advanced glycation end products, vascular endothelial growth factor

## Abstract

Glycation of extracellular matrix proteins has been demonstrated to contribute to the pathogenesis of vascular complications. However, no previous report has shown the role of glycated fibronectin (FN) in vascular endothelial growth factor (VEGF)‐induced angiogenesis. Thus, this study aimed to investigate the effects of glycated FN on VEGF signalling and to clarify the molecular mechanisms involved. FN was incubated with methylglyoxal (MGO) in vitro to synthesize glycated FN, and human umbilical vein endothelial cells (HUVECs) were seeded onto unmodified and MGO‐glycated FN. Then, VEGF‐induced angiogenesis and VEGF‐induced VEGF receptor‐2 (VEGFR‐2) signalling activation were measured. The results demonstrated that normal FN‐positive bands (260 kD) vanished and advanced glycation end products (AGEs) appeared in MGO‐glycated FN and glycated FN clearly changed to a higher molecular mass. The glycation of FN inhibited VEGF‐induced VEGF receptor‐2 (VEGFR‐2), Akt and ERK1/2 activation and VEGF‐induced cell migration, proliferation and tube formation. The glycation of FN also inhibited the recruitment of c‐Src to VEGFR‐2 by sequestering c‐Src through receptor for AGEs (RAGE) and the anti‐RAGE antibody restored VEGF‐induced VEGFR‐2, Akt and ERK1/2 phosphorylation, endothelial cell migration, proliferation and tube formation. Furthermore, the glycation of FN significantly inhibited VEGF‐induced neovascularization in the Matrigel plugs implanted into subcutaneous tissue of mice. Taken together, these data suggest that the glycation of FN may inhibit VEGF signalling and VEGF‐induced angiogenesis by uncoupling VEGFR‐2‐c‐Src interaction. This may provide a novel mechanism for the impaired angiogenesis in diabetic ischaemic diseases.

## 1 INTRODUCTION

Diabetes mellitus, a progressive, chronic disease, is characterized by hyperglycaemia and can cause both microvascular and macrovascular complications.^1^, ^2^ Incompetent angiogenesis generally exists in diabetic vascular complications, and a lot of abnormalities associated with both excessive and defective angiogenesis have been observed in people with diabetes.^3^, ^4^ Angiogenesis is driven by a multiplicity of molecular mechanisms, and vascular endothelial growth factor (VEGF) is well known to be one of the most important angiogenic mediators under both physiological and pathophysiological conditions.^5^, ^6^ In the haemangioblast, VEGF receptor‐2 (VEGFR‐2) is the firstly expressed endothelial tyrosine kinase receptor and targeted blockade of the VEGFR‐2 in mice inhibits endothelial differentiation and blood‐island formation.^7^, ^8^ However, diabetes mellitus is associated with impaired activation of VEGF/VEGFR‐2‐induced signalling pathway, which we now call VEGF resistance.^9^, ^10^ VEGF resistance has been demonstrated to be one of the molecular bases for the abnormal angiogenesis in diabetes mellitus.^11^ However, no previous study has fully illuminated the mechanisms involved in VEGF resistance.

Advanced glycation end products (AGEs), which are formed on macromolecules, in particular, proteins, by non‐enzymatic glycoxidation in hyperglycaemia environment, are well known to contribute to the pathogenesis of vascular complications in diabetes.^12^, ^13^ The extracellular matrix (ECM) proteins can be directly modified by AGEs, and the accumulation of glycated ECM proteins in the vessel wall may promote the vasculature stiffness and impair vascular cell structure and function.^14^, ^15^ Furthermore, the important arginine residues, such as arg‐gly‐asp (RGD) motifs, in ECM proteins may be directly modified by AGEs, causing loss of charge and structural distortion, which is associated with decreased binding affinity of integrins and cell detachment.^16^ Additionally, AGE‐modified ECM can directly bind to receptor for AGEs (RAGE) and activate intracellular pathways.^17^, ^18^ The ECM proteins have been demonstrated to play important roles in regulating VEGF signalling by interaction with integrins. Fibronectin (FN) is one of the components of the ECM proteins and is almost localized into all tissues. In the developing embryo, both FN and its major receptor, integrin α5β1, play a crucial role in vasculogenesis and angiogenesis.^19^, ^20^, ^21^ FN also contributes to the regulation of VEGF signalling and VEGF‐induced angiogenesis. VEGF can directly bind specifically to FN, and the extracellular interaction between FN and VEGF is of crucial importance in VEGF signalling activation.^22^, ^23^ It has been shown that glycation may alter the functions of ECM protein, including vitronectin,^24^ laminin,^16^, ^25^ collagen^26^, ^27^ and FN.^16^, ^17^, ^28^ However, no previous studies have investigated the effects of the glycated FN on VEGF signalling and VEGF‐induced angiogenesis.

It has been demonstrated that c‐Src also plays a crucial role in regulating VEGF signalling. With VEGF stimulation, Src, but not Yes or Fyn, directly interacts with VEGFR‐2, indicating that c‐Src is the main Src family member in the regulation of endothelial cell functions.^29^ VEGF‐induced recruitment of c‐Src to VEGFR‐2 is critical for VEGF angiogenic signalling pathway activation.^30^ On the other hand, the binding of AGEs to RAGE induces a cooperative binding interaction between c‐Src and RAGE.^31^ Therefore, in this study we hypothesized that MGO‐glycated FN may directly bind to RAGE and impair VEGF angiogenic signalling pathway by sequestering c‐Src.

## 2 MATERIALS AND METHODS

### 2.1 Chemicals and reagents

Primary human umbilical vein endothelial cells (HUVECs) were purchased from Cascade Biologics. FN, MGO and su6656 were obtained from Sigma. Recombinant VEGF‐A, recombinant RAGE, recombinant human integrin α5β1 protein, RAGE function‐blocking antibody, biotinylated anti‐RAGE antibody, biotinylated anti‐integrin β1 antibody, streptavidin‐HRP conjugate and HRP substrate were from R&D Systems. Growth‐factor‐reduced Matrigel Matrix was from BD Biosciences. Antibodies to phosphorylated VEGFR‐2, total VEGFR‐2, phosphorylated Akt, total Akt, phosphorylated extracellular regulated protein kinases 1/2 (ERK1/2), total ERK1/2, phosphorylated nuclear factor‐κB (NF‐κB), total NF‐κB and CD31 were from Cell Signaling Technology. Antibodies to FN and AGEs were from Abcam. Antibodies to RAGE and c‐Src were from Santa Cruz Biotechnology. Pierce classic IP kit was from Thermo Scientific.

### 2.2 Cell culture

HUVECs were cultured in Medium 200 (Cascade Biologics) with low‐serum growth supplement, and cells used were passaged 3‐7 times.

### 2.3 Glycation of FN in vitro

As previously described,^16^, ^18^ FN was glycated by incubating the protein (1 mg/mL) with MGO (0.1, 1, 10 and 50 mM) in 100 mM sodium phosphate buffer, at 37°C for 7 days. Unmodified FN was subjected to the same conditions without MGO. To identify the characterization of glycation of FN by MGO, AGE formation was detected by Western blotting and FN was also immunoblotted in the same blots after stripping. Furthermore, AGE‐specific fluorescence at emission of 440 nm and excitation of 370 nm was measured using a fluorescence spectrophotometer.

### 2.4 Cell migration assay

Transwell migration chambers with an 8.0‐μm‐size porous membrane were used to perform HUVEC migration. HUVECs (2 × 10^4^) were added to the upper chambers, which were pre‐coated with FN or MGO‐FN overnight at 4°C. Then, the cells were stimulated with VEGF (50 ng/mL) or vehicle control for 24 hours. If there is a need, cells would be pre‐treated with RAGE function‐blocking antibody (10 μg/mL) or Src inhibitor, su6656 (5 μM) for 30 minutes before VEGF (50 ng/mL) stimulation. Then, cells remaining in the upper chamber were removed and cells that had migrated to the lower chamber were stained with crystal violet (0.5%) and counted.

### 2.5 Tube formation assay

HUVECs (1 × 10^5^) were seeded onto growth‐factor‐reduced Matrigel (250 μL) with FN or MGO‐glycated FN in a 24‐well plate and stimulated by VEGF (50 ng/mL) or vehicle control for 24 hours. In some experiments, cells were pre‐treated with RAGE function‐blocking antibody (10 μg/mL) or Src inhibitor, su6656 (5 μM) for 30 minutes, followed by seeding onto Matrigel. Then, tube formation was photographed by an EVOS microscope (Electron Microscopy Sciences) and the total length of the tube was quantified using Image‐Pro Plus software.

### 2.6 Cell proliferation assay

HUVECs (1 × 10^5^) were cultured on 96‐well plates, which were pre‐coated with FN or MGO‐FN overnight at 4°C, and grown to 50% confluence. Then, the cells were stimulated with VEGF or vehicle control for 24 hours. In some experiments, cells were pre‐treated with RAGE function‐blocking antibody (10 μg/mL) for 30 minutes before VEGF (50 ng/mL) stimulation. Then, a 10‐μL aliquot of Cell Counting Kit 8 (CCK8) solution was added to each well for 4 hours. The absorbance was measured at 450 nm using a Spectra Max M5 microplate reader (Molecular Devices). HUVEC proliferation was evaluated based on optical density (OD) values.

### 2.7 Western blotting

HUVECs were cultured on FN or MGO‐glycated FN and grown to confluence, followed by stimulation with VEGF (50 ng/mL) for 10 minutes. Then, the cells were lysed in RIPA lysis buffer supplemented with 1% (v/v) protease and phosphatase inhibitor cocktail (Thermo Scientific). Equal amounts of proteins were subjected to SDS‐PAGE and transferred to polyvinylidene fluoride membranes (Bio‐Rad Laboratories). Blots were blocked with non‐fat milk solution (5%) for 60 minutes at room temperature and incubated with primary antibodies for phosphorylated VEGFR‐2 (1:1000), phosphorylated Akt (1:1000) and phosphorylated ERK1/2 (1:2000) overnight at 4°C. After stripping, the same membranes were probed with antibodies against VEGFR‐2 (1:1000), Akt (1:1000) and ERK1/2 (1:2000) to detect VEGFR‐2, Akt and ERK1/2 signalling. Then, the blots were detected using HRP‐conjugated species‐specific respective goat IgG (Santa Cruz Biotechnology) for 60 minutes at room temperature. The protein bands were visualized with chemiluminescence, and ImageJ software was used to measure the density of the bands.

### 2.8 Immunoprecipitation

HUVECs cultured on FN or MGO‐FN were exposed to VEGF (50 ng/mL) for 10 minutes. Classic IP kit was used to perform protein immunoprecipitation. The samples were incubated with protein A/G plus agarose beads and primary antibodies against c‐Src, VEGFR‐2 or RAGE overnight at 4°C, with gentle rotation. According to the IP kit instruction, the immune complexes were captured and solubilized by 50 μL SDS sample buffer. Then, the immune complexes were immunoblotted with primary antibodies against VEGFR‐2 (1:500), c‐Src (1:250) and RAGE (1:250).

### 2.9 Solid‐phase binding assay

Solid‐phase binding assay was used to investigate the potential binding of unmodified FN and glycated FN to recombinant RAGE or recombinant integrin α5β1 in vitro. Microliter plate wells were coated with unmodified or glycated FN at 4°C overnight. After washing and blocking, recombinant RAGE or recombinant integrin α5β1 was added and incubated at room temperature for 1 hour, after which biotinylated anti‐RAGE antibody or biotinylated anti‐β1 antibody, streptavidin‐HRP conjugate and HRP substrate were sequentially added and OD450 was measured.

### 2.10 In vivo angiogenesis assay

Matrigel plug experiments were used to investigate the angiogenesis in vivo. All animal care and experimental procedures were approved by the Animal Care and Use Committee of Southwest Medical University. Growth‐factor‐reduced Matrigel (500 μL) was pre‐mixed with 30 units of heparin and one of the following: (a) FN (10 μg/mL), (b) FN and VEGF (250 ng/mL), and (c) MGO‐FN (10 μg/mL) or MGO‐FN and VEGF. Then, the mixed Matrigel was injected into subcutaneous tissue of 8‐week‐old C57BL/6J mice. After 14 days, Matrigel plugs were harvested, fixed, embedded in paraffin and sectioned. Then, 4‐μm paraffin sections were incubated with antibody directed against CD31 at 4°C overnight, following by incubation with secondary antibody for 60 minutes at 37°C. Then, the samples were performed with DAB substrate working solution and CD31‐positive infiltrating microvessels were calculated in five microscopic fields using Image‐Pro Plus software.

### 2.11 Statistical analysis

All of the data are shown as mean ± standard deviation (SD). Results were analysed by one‐way ANOVA followed by post hoc comparison. A *P* value of <.05 was considered as statistically significant.

## 3 RESULTS

### 3.1 Glycation of FN by MGO

**FIGURE 1:**
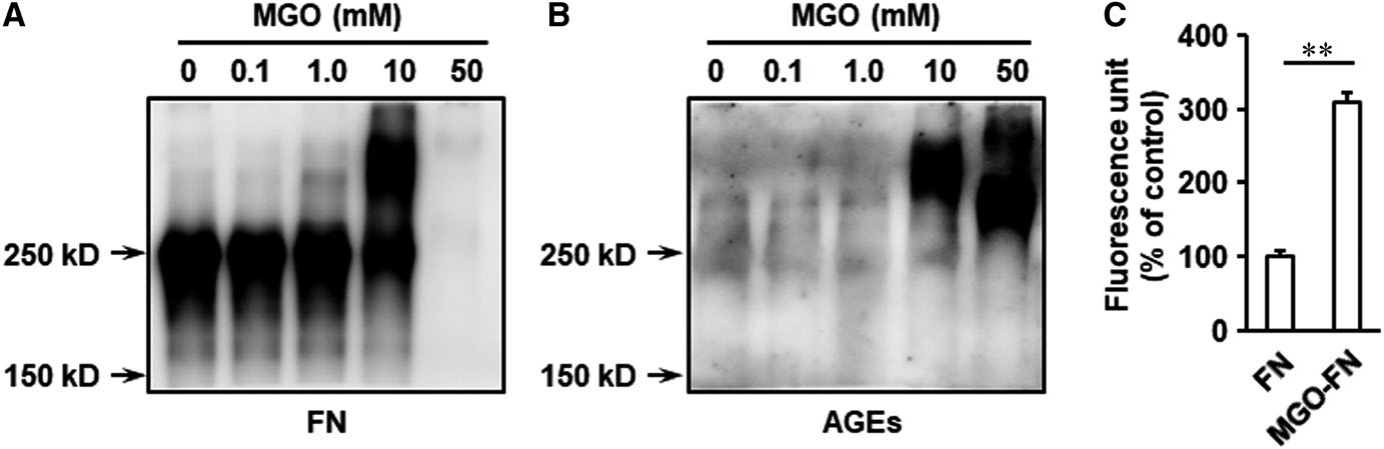
Characterization of glycation of FN by MGO. FN (1 mg/mL) was incubated with MGO (0, 0.1, 1, 10 and 50 mM) at 37°C for 7 days. A, The samples were separated by SDS‐PAGE, and FN was detected with immunoblotting. B, AGEs were also immunoblotted on the same blots after stripping. C, The fluorescence intensity of MGO‐FN (50 mM MGO) was measured at 370/440 nm in the fractions. Results represent the mean ± SD for triplicate determinations. **P < .01

To model diabetes‐induced alteration of FN in vitro, FN was incubated with MGO (0, 0.1, 1.0, 10 and 50 mM), which is formed during anaerobic glycolysis and mediates extracellular matrix glycation, for 7 days at 37°C. To explore the characterization of MGO‐glycated FN, the incubates were analysed by Western blotting using anti‐FN antibody and anti‐AGEs antibody in the same membrane. The results demonstrated that 1.0 and 10 mM MGO induced the formation of higher molecular mass FN molecules (Figure 1A), indicating the changes in glycosylation and the existence of covalently cross‐linked products. Though the normal FN‐positive band completely vanished in FN in the presence of 50 mM MGO, the AGE bands clearly appeared in FN incubated with 10 and 50 mM MGO (Figure 1B), which suggested that high concentration of MGO may change the conformation of FN and induce glycated FN formation. To further identify the production of glycated FN, AGE‐specific fluorescence at an excitation of 370 nm and an emission of 440 nm was measured. In agreement with the Western blotting results, the fluorescence of 50 mM MGO modified FN was significantly increased (Figure 1C), which indicated that glycated FN had been successfully formed in vitro.

### 3.2 Glycated FN inhibits VEGF signalling and VEGF‐induced cell migration, proliferation and tube formation

**FIGURE 2:**
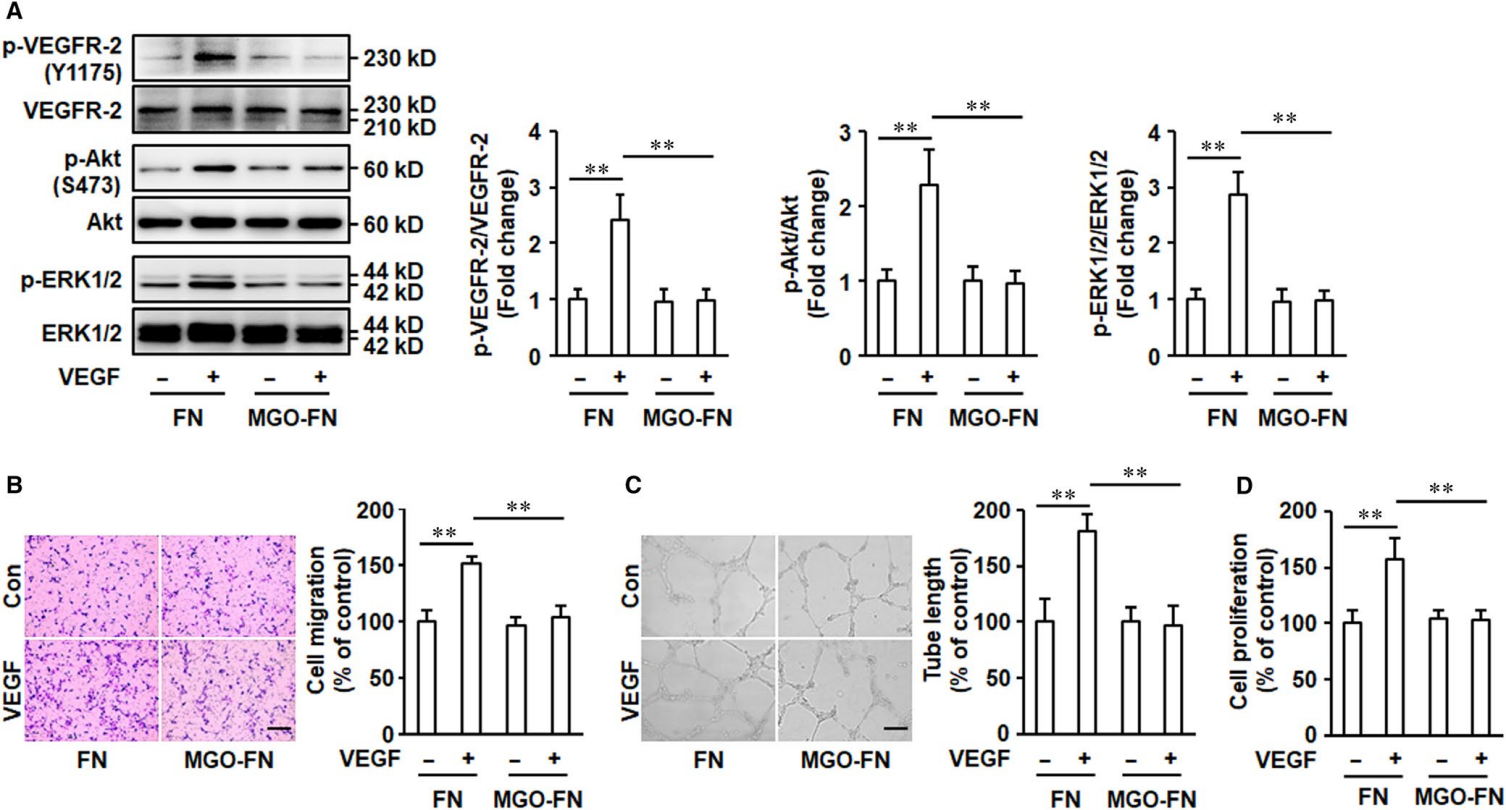
Glycation of FN inhibits VEGF signalling and VEGF‐induced angiogenesis. A, MGO‐FN inhibits VEGF‐induced activation of VEGFR‐2. HUVECs were cultured on FN or MGO‐FN and stimulated with VEGF (50 ng/mL) or vehicle control for 10 minutes. Phosphorylation (p) of VEGFR‐2, Akt and ERK1/2, and total VEGFR‐2, Akt and ERK1/2 were analysed by Western blotting in total cell lysates. Representative images of three independent experiments and densitometric analysis of phosphorylated VEGFR‐2, Akt and ERK1/2 normalized to total VEGFR‐2, Akt and ERK1/2 are shown. All data shown are mean ± SD for triplicate experiments and are expressed as fold changes. **P < .01. B, MGO‐FN inhibits VEGF‐induced cell migration. HUVECs were added to the upper chambers containing FN or MGO‐FN‐coated porous filters and then exposed to VEGF (50 ng/mL) or vehicle control. After 24 hours, the cells were fixed, stained with crystal violet and the cells that had migrated to the lower chambers were counted. Representative images of cell migration are shown. Scale bar is 200 μm. Quantitative assessment of triplicate cell migration experiments was performed. All data shown are mean ± SD and are expressed as % of control. **P < .01. C, MGO‐FN inhibits VEGF‐induced tube formation. HUVECs were seeded onto Matrigel with FN or MGO‐FN in the presence of VEGF (50 ng/mL) or vehicle control for 16 hours. Representative images of tube formation are shown. Scale bar is 500 μm. Quantitative assessment of triplicate tube formation experiments was performed. All data shown are mean ± SD and are expressed as % of control. **P < .01. D, MGO‐FN inhibits VEGF‐induced endothelial cell proliferation. HUVECs were cultured on FN‐ or MGO‐FN‐coated 96‐well plates and grown to 50% confluence, followed by stimulation with VEGF or vehicle control for 24 hours. Then, cell proliferation was measured using the CCK8 assay. All data shown are mean ± SD for triplicate experiments and are expressed as % of control. **P < .01

FN significantly amplifies VEGF signalling and VEGF‐mediated endothelial cell activation.^22^, ^23^ To detect the roles of glycated FN in activation of VEGF signalling, HUVECs grown on control FN or MGO‐glycated FN were stimulated with VEGF for 10 minutes. The results showed that the phosphorylation of VEGFR‐2 significantly increased with VEGF stimulation in HUVECs cultured on FN. However, VEGF‐induced VEGFR‐2 activation was inhibited, when the cells were cultured on MGO‐glycated FN (Figure 2A). The downstream angiogenic signalling of VEGF/VEGFR‐2, such as Akt and ERK1/2, was further measured, and glycated FN also significantly inhibited VEGF‐evoked Akt and ERK1/2 phosphorylation (Figure 2A). We also investigated the effects of glycated FN on the expression of VEGFR‐2 and VEGF‐induced activation of VEGFR‐2 signalling pathway in a longer time manner. The results showed glycation of FN did not significantly change total VEGFR‐2 expression when HUVECs were cultured on MGO‐FN for 24 and 48 hours (Figure S1). Furthermore, with VEGF (50 ng/mL) stimulation for 24 and 48 hours, the phosphorylation of VEGFR‐2, Akt and ERK1/2 has not been activated and showed no significant difference among the six groups (Figure S2). This most probably because VEGF rapidly induced activation of VEGFR‐2 and the phosphorylation of VEGFR‐2 decreased to the normal level under longer time stimulation.

We further identified the effects of glycated FN on VEGF‐activated endothelial cell physiological responses. HUVECs were plated onto the upper transwell migration chambers pre‐coated with control FN or MGO‐glycated FN, followed by VEGF stimulation for 24 hours. The results demonstrated that VEGF significantly promoted migration of HUVECs cultured on control FN. However, when the cells were cultured on MGO‐glycated FN, VEGF‐induced increase of HUVEC migration was significantly inhibited (Figure 2B). In endothelial cells, VEGF also induces formation of cordlike structures that mimic blood vessels. To investigate the effects of glycated FN on tube formation, HUVECs were added to 24‐well plates pre‐coated with Matrigel in the presence of control FN or glycated FN and were exposed to VEGF. The results showed the glycation of FN also significantly inhibited the augmentation of tube formation induced by VEGF (Figure 2C). Furthermore, VEGF‐induced endothelial cell proliferation was also significantly inhibited by glycation of FN (Figure 2D). Taken together, these data suggested that MGO‐glycated FN impairs activation of VEGF pro‐angiogenic signalling in endothelial cells.

### 3.3 Glycated FN inhibits VEGF‐induced VEGFR‐2‐c‐Src interaction

**FIGURE 3:**
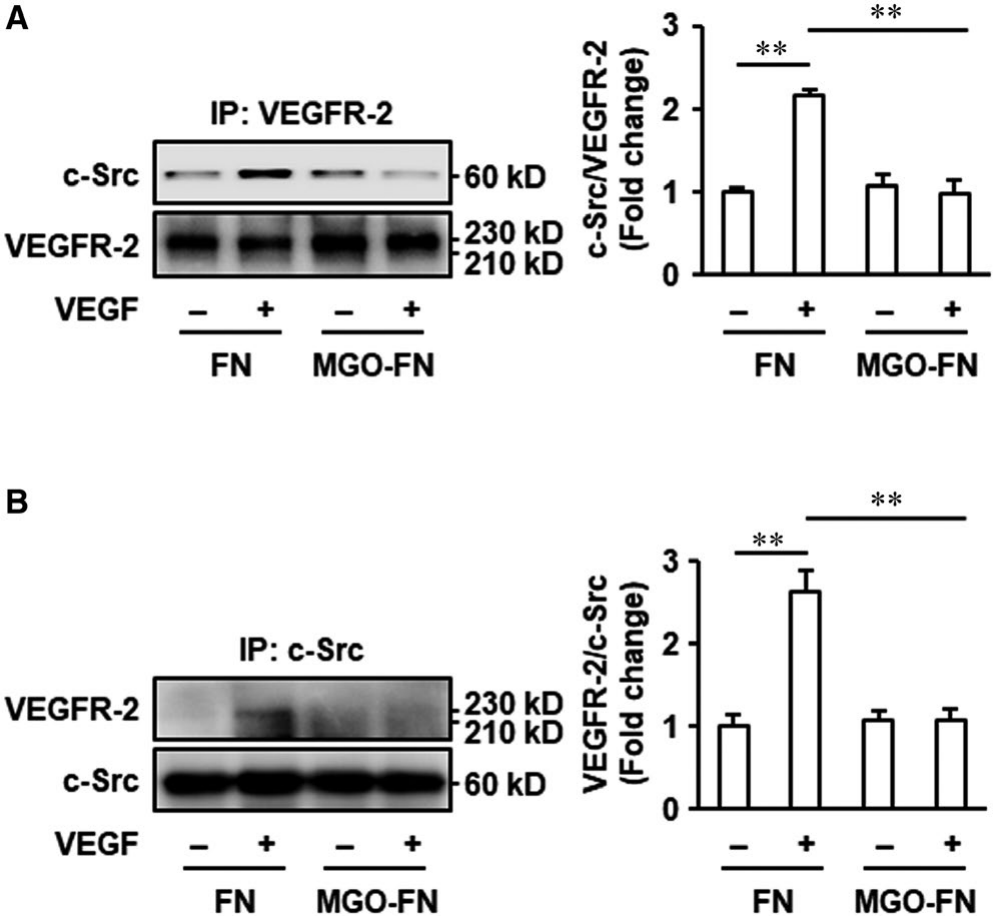
Glycation of FN inhibits VEGF‐induced VEGFR‐2‐c‐Src interaction. HUVECs were cultured on FN or MGO‐FN and stimulated with VEGF (50 ng/mL) or vehicle control for 10 minutes. A, Cell lysates were prepared and immunoprecipitated with anti‐VEGFR‐2 antibody and protein A/G plus agarose beads. B, Cell lysates were immunoprecipitated with anti‐c‐Src antibody and protein A/G plus agarose beads. Then, captured proteins were analysed by Western blotting with anti‐c‐Src and anti‐VEGFR‐2 antibodies as indicated. Representative images of three independent experiments and densitometric analysis are shown. All data shown are mean ± SD for triplicate experiments and are expressed as fold changes. **P < .01

VEGFR‐2‐c‐Src interaction plays an important role in VEGF signalling activation and VEGF‐induced pro‐angiogenic responses in endothelia cells.^29^, ^30^ Therefore, the effects of MGO‐glycated FN on VEGF‐induced VEGFR‐2‐c‐Src interaction were determined. VEGFR‐2‐c‐Src complexes were captured by an immobilized anti‐VEGFR‐2 antibody (Figure 3A) or an anti‐c‐Src antibody (Figure 3B), respectively, and detected by immunoblotting. Both of the results demonstrated the VEGFR‐2‐c‐Src coimmunoprecipitation was significantly enhanced in HUVECs cultured on unmodified FN, but there was no increase in cells cultured on MGO‐glycated FN (Figure 3). These data indicated that VEGF leads to the VEGFR‐2‐c‐Src complex formation and that the glycation of FN inhibits this process.

The effects of blockade of c‐Src on VEGF‐evoked pro‐angiogenic responses in endothelial cells were further examined. HUVECs plated onto unmodified FN were pre‐treated with the Src inhibitor su6656 for 30 minutes before VEGF treatment. The results showed that su6656 significantly inhibited VEGF‐induced endothelial cell migration and tube formation (Figure S3). Taken together, these results and the immunoprecipitation experiments indicated that glycation of FN‐induced VEGF resistance may be associated with the inhibition of VEGFR‐2‐c‐Src interaction stimulated by VEGF.

### 3.4 Glycated FN directly binds to RAGE

**FIGURE 4:**
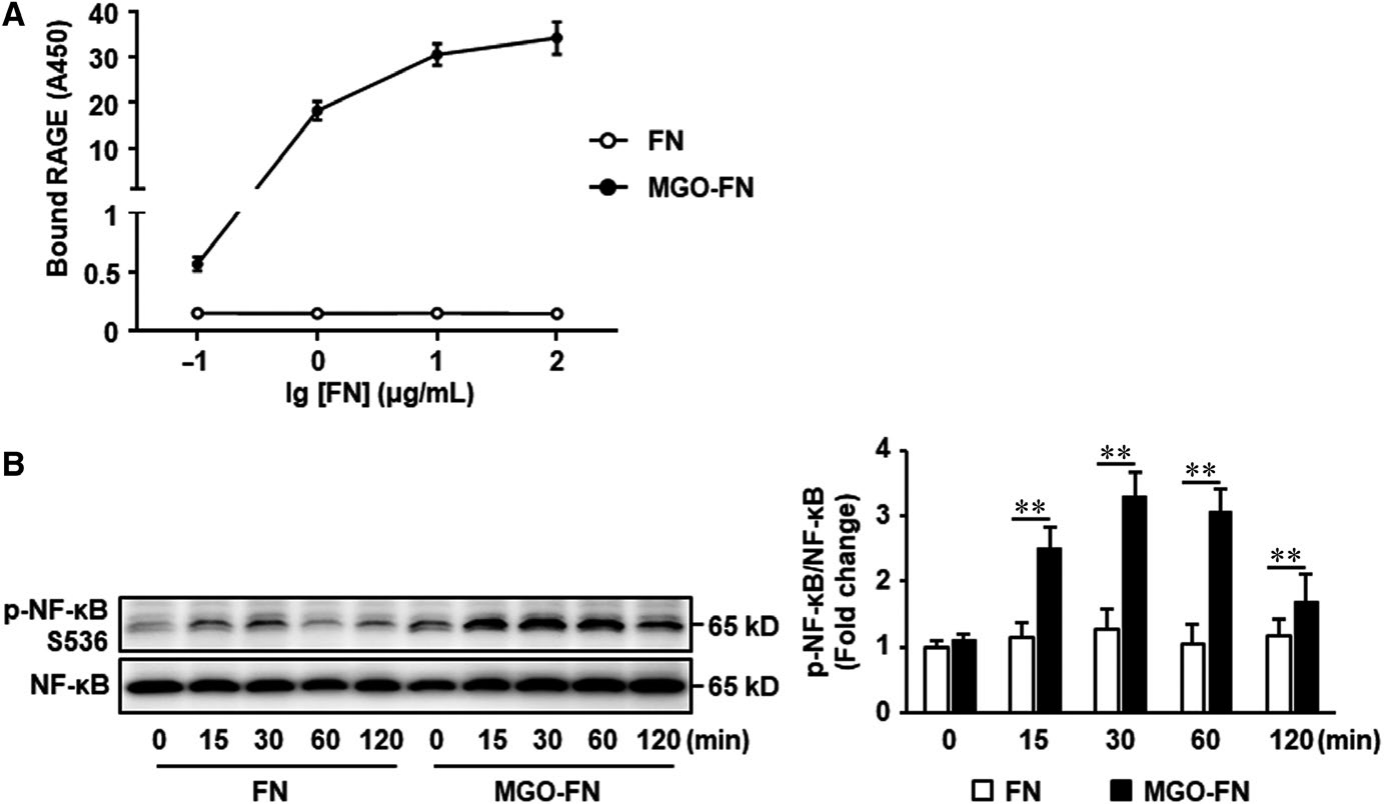
Glycated FN directly binds to RAGE. A, 96‐well plates were coated with FN or MGO‐FN at 4°C overnight. After washing and blocking, recombinant RAGE (5 μg/mL) was added and incubated at 37°C for 1 hour. Bound RAGE was detected by sequential addition of biotinylated anti‐RAGE antibody, streptavidin‐HRP conjugate and HRP substrate and measurement of OD450. Data shown are mean ± SD for triplicate experiments. B, HUVECs were seeded onto FN or MGO‐FN for 0, 15, 30, 60 and 120 minutes. Phosphorylation (p) of NF‐κB and total NF‐κB were analysed by Western blotting in total cell lysates. Representative images of three independent experiments and densitometric analysis of phosphorylated NF‐κB normalized to total NF‐κB are shown. All data shown are mean ± SD for triplicate experiments and are expressed as fold changes. **P < .01

To further investigate how glycation of FN prevents the binding of c‐Src to VEGFR‐2, we hypothesized that glycated FN directly binds to receptor for AGEs (RAGE), as AGEs can induce formation of RAGE‐c‐Src complexes.^31^ Solid‐phase binding assay was used to detect the potential binding of glycated FN to recombinant RAGE in vitro. The results showed that unmodified FN did not bind to RAGE, but glycated FN bound to RAGE in a concentration‐dependent manner (Figure 4A). However in the meanwhile, the normal binding between FN and integrin α5β1 was significantly inhibited by the glycation (Figure S4). To further confirm the directly binding of glycated FN to RAGE, the downstream signalling pathway of RAGE, such as NF‐κB, was determined in endothelial cells. The results demonstrated that the NF‐κB phosphorylation was significantly increased in HUVECs plated onto glycated FN (Figure 4B). All of these results suggested glycation of FN may directly bind to RAGE in endothelial cells.

### 3.5 A pool of c‐Src is sequestered by RAGE upon glycated FN stimulation

**FIGURE 5:**
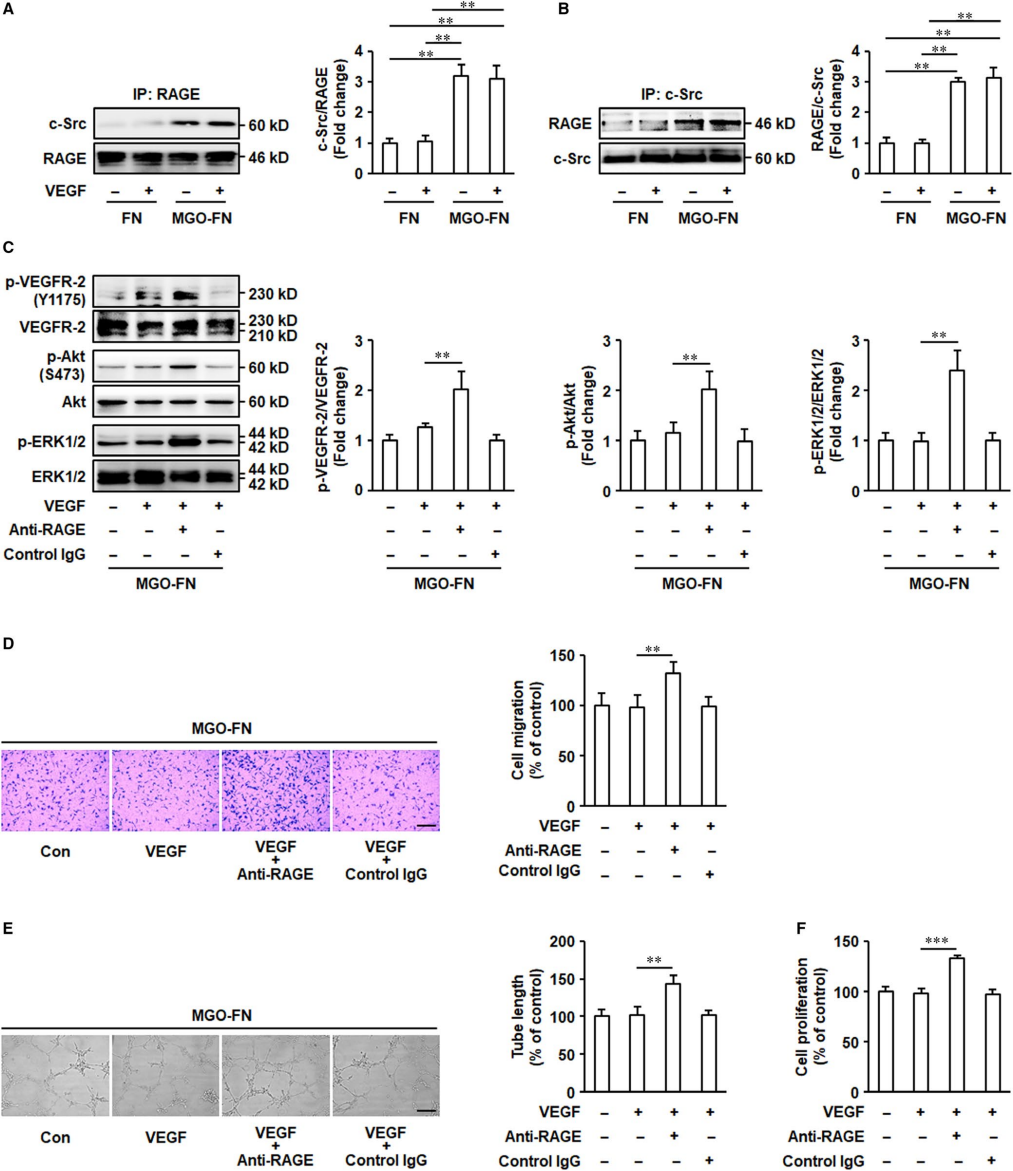
A pool of c‐Src is sequestered by RAGE upon glycated FN stimulation. A and B, Glycation of FN induces RAGE‐c‐Src complex formation. MGO‐FN induces RAGE‐c‐Src interaction. HUVECs were cultured on FN or MGO‐FN and stimulated with VEGF (50 ng/mL) or vehicle control for 10 minutes. Cell lysates were prepared and immunoprecipitated with anti‐RAGE or anti‐c‐Src antibodies and protein A/G plus agarose beads. Then, captured proteins were analysed by Western blotting with anti‐c‐Src and anti‐RAGE antibodies as indicated. Representative images of three independent experiments and densitometric analysis are shown. All data shown are mean ± SD for triplicate experiments and are expressed as fold changes. **P < .01. C, The blockade of RAGE restores activation of VEGF signalling. HUVECs were pre‐treated with anti‐RAGE antibody (10 μg/mL) or control IgG for 30 minutes. Then, cells were seeded onto MGO‐FN‐coated 6‐well plates, followed by stimulation with VEGF (50 ng/mL) or vehicle control for 10 minutes. Phosphorylation (p) of VEGFR‐2, Akt and ERK1/2, and total VEGFR‐2, Akt and ERK1/2 were analysed by Western blotting in total cell lysates. Representative images of three independent experiments and densitometric analysis of phosphorylated VEGFR‐2, Akt and ERK1/2 normalized to total VEGFR‐2, Akt and ERK1/2 are shown. All data shown are mean ± SD for triplicate experiments and are expressed as fold changes. **P < .01. D, The blockade of RAGE restores VEGF‐induced cell migration. HUVECs were pre‐treated with anti‐RAGE antibody (10 μg/mL) or control IgG for 30 minutes. Then, cells were added to the upper chambers containing MGO‐FN‐coated porous filters, followed by stimulation with VEGF (50 ng/mL) or vehicle control. After 24 hours, the cells were fixed, stained with crystal violet and the cells that had migrated to the lower chambers were counted. Representative images of cell migration are shown. Scale bar is 200 μm. Quantitative assessment of triplicate cell migration experiments was performed. All data shown are mean ± SD and are expressed as % of control. **P < .01. E, The blockade of RAGE restores VEGF‐induced tube formation. HUVECs, pre‐treated with anti‐RAGE antibody (10 μg/mL) or control IgG for 30 minutes, were seeded onto Matrigel with MGO‐FN in the presence of VEGF (50 ng/mL) or vehicle control for 24 hours. Representative images of tube formation are shown. Scale bar is 500 μm. Quantitative assessment of triplicate tube formation experiments was performed. All data shown are mean ± SD and are expressed as % of control. **P < .01. F, The blockade of RAGE restores VEGF‐induced cell proliferation. HUVECs, pre‐treated with anti‐RAGE antibody (10 μg/mL) or control IgG for 30 minutes, were seeded onto MGO‐FN‐coated 96‐well plates and grown to 50% confluence, followed by stimulation with VEGF or vehicle control for 24 hours. Then, cell proliferation was measured using the CCK8 assay. All data shown are mean ± SD for triplicate experiments and are expressed as % of control. ***P < .001

To investigate the effects of binding of glycated FN to RAGE on c‐Src, the interaction between RAGE and c‐Src was measured. RAGE‐c‐Src complexes were captured by an immobilized anti‐RAGE antibody (Figure 5A) or an anti‐c‐Src antibody (Figure 5B), respectively, and detected by immunoblotting. The results demonstrated that no matter with or without VEGF stimulation, the formation of RAGE‐c‐Src complexes was significantly increased in HUVECs grown on glycated FN (Figure 5A,B). Concomitant with this increase, the accumulation of VEGFR‐2‐c‐Src complexes upon VEGF stimulation was significantly decreased in cells grown on glycated FN (Figure 3). This suggests that glycated FN‐induced RAGE‐c‐Src interaction can compete for binding to c‐Src with VEGF‐stimulated VEGFR‐2 and therefore that RAGE may limit the pool of Src available for interaction with VEGFR‐2.

Remarkably, the effects of blockade of RAGE on VEGF signalling activation were further determined. HUVECs plated onto glycated FN were exposed to the anti‐RAGE blocking antibody for 60 minutes, followed by VEGF stimulation. The results showed that the pre‐treatment with anti‐RAGE antibody restored phosphorylation of VEGFR‐2, Akt and ERK1/2 (Figure 5C) and VEGF‐induced angiogenesis in vitro (Figure 5D‐F), indicating that the blockade of RAGE eliminates the inhibitory effects of MGO‐glycated FN on VEGF angiogenic signalling. As a whole, these data indicate that glycation of FN can directly bind to RAGE and result in the interaction of RAGE to c‐Src, thereby preventing the recruitment of c‐Src to VEGFR‐2 and the consequent activation of VEGF‐VEGFR‐2 signalling pathway that involves Akt and ERK1/2 that induces endothelial cell migration and tube formation.

### 3.6 Glycated FN inhibits VEGF‐induced angiogenesis in vivo

**FIGURE 6:**
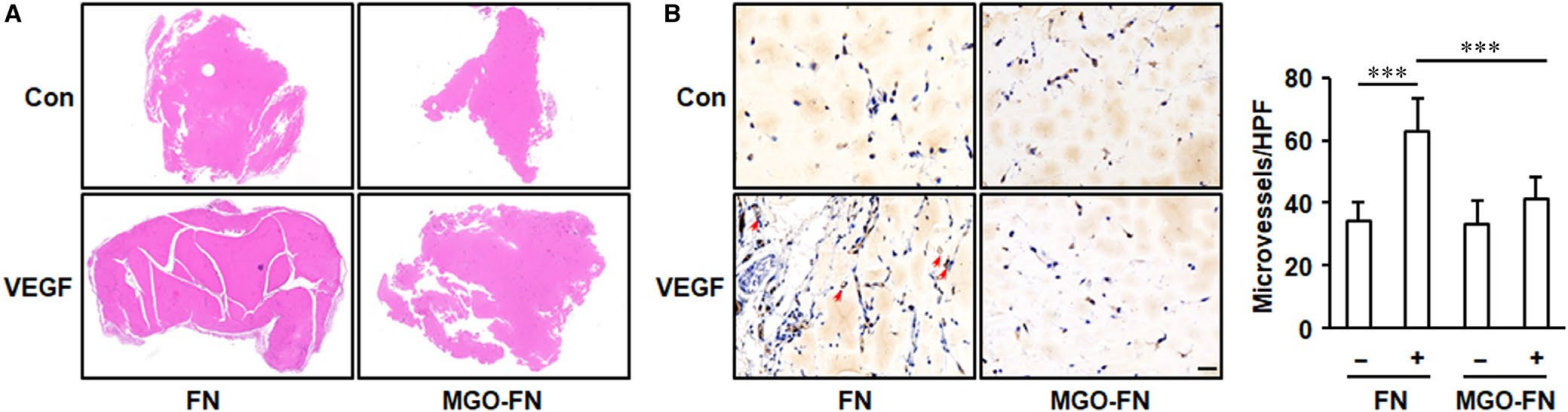
Glycation of FN inhibits VEGF‐induced angiogenesis in Matrigel plug assay. Matrigel supplemented with FN, FN and VEGF, MGO‐FN or MGO‐FN and VEGF was injected subcutaneously in mice for 14 days. Then, the Matrigel plugs were harvested, fixed and sectioned. A, The plugs were stained with haematoxylin and eosin, and the representative images are shown. B, The endothelial cells were labelled by immunochemical staining of CD31, and the representative images of CD31‐positive microvessels (arrows) in Matrigel plugs are shown. Scale bar is 100 μm. Quantitative assessment of microvessel formation was performed, and all data shown are mean ± SD. ***P < .001

To investigate the effects of glycated FN on VEGF‐induced angiogenesis under physiological conditions, growth‐factor‐reduced Matrigel impregnated with VEGF and FN or MGO‐FN was injected into subcutaneous of mice. After 2 weeks, the solidified gels were harvested and blood vessel invasion into them was evaluated by CD31 staining. VEGF significantly increased endothelial cell invasion into Matrigels mixed with FN. However, when the Matrigels were mixed with MGO‐FN, VEGF‐induced neovascularization was significantly inhibited (Figure 6). These data clarify the significance of anti‐angiogenesis effects of MGO‐glycated FN in vivo.

## 4 DISCUSSION

During diabetes mellitus, AGEs, which are well known to contribute to vascular complications, accumulate in the vessel wall and increase ECM protein glycation and cross‐linking. Modification of proteins by AGEs constitutes one of the key pathogenic events in diabetes and accumulation of these adducts with FN can be about as much as four times as other ECM proteins.^32^ Previous studies indicate that the glycation of FN has been proposed to contribute to diabetic microangiopathy evolution.^28^ Although it is well known diabetes‐induced modification of FN leads to impaired endothelial function and differentiation,^33^, ^34^ it is not fully clarified what results in glycated FN accumulation and how glycated FN exerts its antiangiogenic effects.

MGO is significantly increased in diabetic serum and as a precursor for AGE formation, can lead to rapid modification of protein amino acid residues.^35^, ^36^ Previous studies have demonstrated that MGO‐derived AGEs develop rapidly on the diabetic mice vessels.^37^ In the present report, we synthesized MGO‐glycated FN and evaluated the conformational and structural properties of MGO modified FN. Consistent with our findings, a previous report also showed the conformational changes in MGO‐glycated FN which existed as high‐molecular‐weight products.^38^ Using tandem mass spectrometry, previous studies have also demonstrated that after treatment with MGO, three lysine and eleven arginine residues in the III_9‐10_ sequence of FN were glycated by AGE modification, indicating the domains which bind to integrin and heparin were AGE modification targets.^18^ Our data and the published studies suggest that characterized changed structure of glycation on FN leads to the loss of interaction between RGD‐binding integrins and their ligands.

Extracellular interaction between FN and VEGF potentiates VEGF signalling and VEGF‐induced angiogenesis. Using a solid‐phase assay, previous reports showed that FN, but not collagen specifically bound to VEGF, and this interaction could localize VEGF, which enhanced VEGF signalling activation and endothelial cell migration.^22^, ^23^ The data in this study demonstrated significant impairment in VEGFR‐2, Akt and ERK1/2 phosphorylation, cell migration, proliferation and tube formation in HUVECs cultured on MGO‐glycated FN, indicating a serious inhibitory effects of glycated FN on VEGF signalling. Our findings suggest that the pro‐angiogenic effects of FN were lost by conformational changes in FN‐VEGF binding under glycosylation. Alternatively, FN can also interact with other ECM proteins, such as collagens, and these interactions contribute to FN‐regulated cell adhesion and migration. Previous studies showed that the modification by AGE decreased the cooperative interactions between FN, heparin and collagens.^32^, ^39^ Therefore, the presence of glycosylation on FN residues in binding sites decreases its binding activity for those proteins thus resulting in a serious impairment in cell adhesion and migration, indicating that the physical binding between VEGF and FN is required for VEGF pro‐angiogenic effects.

Consistent with previous reports which showed that MGO treatment of FN strongly increased glycation,^16^, ^28^, ^40^ our experiments involving immunoblotting and AGE‐specific fluorescence analysis of MGO/FN incubates also demonstrated significantly formation of AGEs. MGO‐glycated FN has been shown to interact with RAGE in vascular smooth muscle cells and NIH 3T3 cells.^17^, ^18^ Similar to these reports, we observed directly binding between MGO‐glycated FN and RAGE using a solid‐phase assay and significantly phosphorylation of NF‐κB in HUVECs. The AGEs‐RAGE binding has been shown to activate many intracellular signalling, including inducing a cooperative binding interaction between c‐Src and RAGE^31^ which is in agreement with the immunoprecipitation results in the present study. However, previous studies^30^ and our experiments involving the blockade of c‐Src showed that c‐Src‐VEGFR‐2 crosstalk is critical for phosphorylation of VEGFR‐2 and activation of the downstream angiogenic signalling pathways. The results in this study demonstrated that MGO‐glycated FN significantly inhibited VEGF‐stimulated recruitment of c‐Src to VEGFR‐2, which is an obligatory process of the pathway by which VEGF provokes angiogenesis, suggesting that the glycation of FN directly uncouples VEGFR‐2‐c‐Src crosstalk and down‐regulates VEGFR‐2 activation. Remarkably, the blockade of RAGE eliminates the inhibitory effects of glycation of FN on VEGF signalling activation and VEGF‐induced angiogenesis. Although few previous studies have focused the role of RAGE in VEGF angiogenic function, a lot of reports have shown that knockout of RAGE or using soluble RAGE significantly increased hindlimb ischaemia‐induced angiogenesis in diabetes animal model,^41^, ^42^, ^43^ which were at least partly consistent with the findings in this study. As a whole, the present results indicate that a pool of c‐Src is sequestered by RAGE upon MGO‐glycated FN stimulation, consequently uncoupling VEGFR‐2‐c‐Src interaction and inhibiting VEGF signalling.

Consistent with the cell culture results, the Matrigel plug experiments also showed significant anti‐angiogenesis effects of glycated FN in vivo. However, a limitation of our Matrigel plug assay is that we cannot definitively proved the inhibitory effects of glycated FN on activation of VEGF‐VEGFR‐2 signalling pathway. Nevertheless, our Matrigel plug assay data complement and support the significance of our in vitro experiments, which demonstrated that glycation of FN inhibits VEGF‐induced angiogenesis. Further in vivo studies will be needed to better clarify the significance of our newly reported regulation mechanism on VEGF‐induced angiogenic signalling in other diseases models.

**FIGURE 7:**
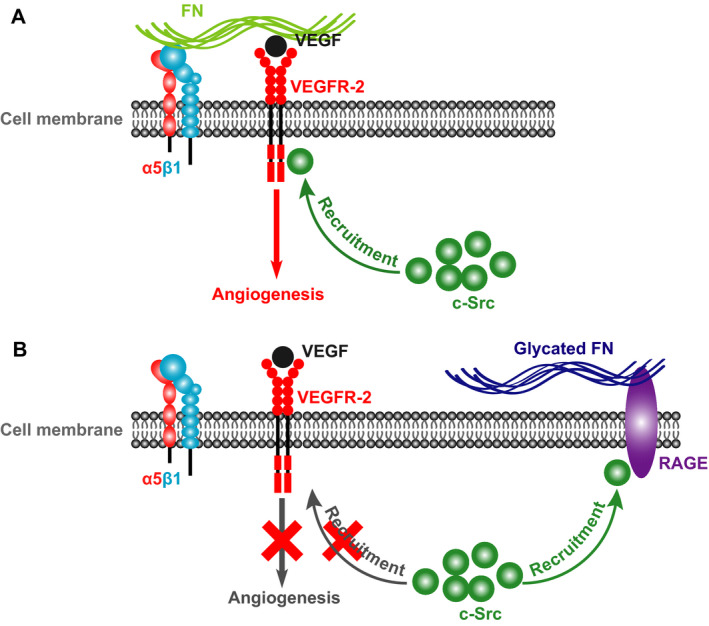
Schematic diagrams representing the molecular mechanism underlying the glycation of FN‐regulated VEGF signalling and VEGF‐induced angiogenesis. A, Under the physiology condition, the binding of FN to VEGF enhances VEGF signalling and VEGF‐induced angiogenesis and the recruitment of c‐Src to VEGFR‐2 is critical for VEGFR‐2 phosphorylation and the downstream angiogenic signalling activation. B, Glycation of FN directly binds to RAGE and results in the interaction of RAGE to c‐Src, thereby preventing the binding of c‐Src to VEGFR‐2 and the consequent activation of VEGF‐VEGFR‐2 signalling pathway that leads to angiogenesis

In summary, the present study showed that MGO‐glycated FN impairs VEGF angiogenic effects with mechanisms involving the inactivation of the VEGF/VEGFR‐2 angiogenic signalling pathway and disruption of the VEGFR‐2‐c‐Src crosstalk (Figure 7). The findings suggest that the formation of glycated FN may be involved in the underlying molecular mechanism of diabetic microangiopathy.

## CONFLICTS OF INTEREST

The authors declare no conflict of interests.

## AUTHOR CONTRIBUTION

**Tangting Chen**: Funding acquisition (lead); Investigation (equal); Methodology (equal). **Jinling Dong**: Investigation (equal). **Haiyan Zhou**: Investigation (lead). **Xin Deng**: Investigation (supporting). **Rong Li**: Investigation (supporting); Methodology (supporting). **Ni Chen**: Investigation (supporting); Methodology (supporting). **Mao Luo**: Formal analysis (equal); Software (equal). **Yongjie Li**: Formal analysis (equal); Software (equal). **Jianbo Wu**: Project administration (equal); Supervision (equal); Writing‐review & editing (equal). **Liqun Wang**: Data curation (equal); Funding acquisition (equal); Project administration (equal); Supervision (equal); Writing‐original draft (equal); Writing‐review & editing (equal).

## Supporting information

Supplementary Material

## Data Availability

The data that support the findings of this study are available from the corresponding author upon reasonable request.
