# Supplementary material for: Glycation of fibronectin inhibits VEGF‐induced angiogenesis by uncoupling VEGF receptor‐2‐c‐Src crosstalk
Source: J Cell Mol Med. 2020 Jul 1;24(16):9154–64. doi: 10.1111/jcmm.15552 (PMC7417727; doi:10.1111/jcmm.15552)
Supplement: Supplementary file 1 — Supplementary Material [file JCMM-24-9154-s001.docx]

**
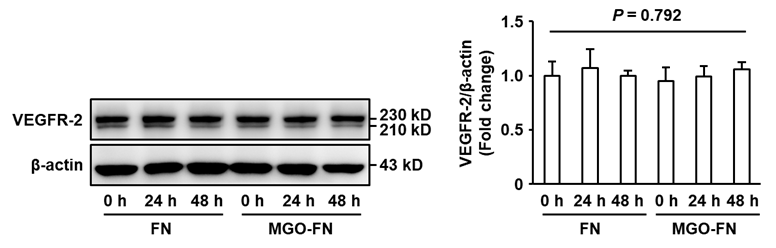
**

**FIGURE S1** The effects of glycated FN on VEGFR-2 expression in a long time manner. HUVECs were cultured on FN or MGO-FN for 24 and 48 h. The expression of total VEGFR-2 was analyzed by western blotting. Representative images of three independent experiments and densitometric analysis of VEGFR-2 normalized to β-actin are shown. All data shown are mean ± SD for triplicate experiments and are expressed as fold changes.

**
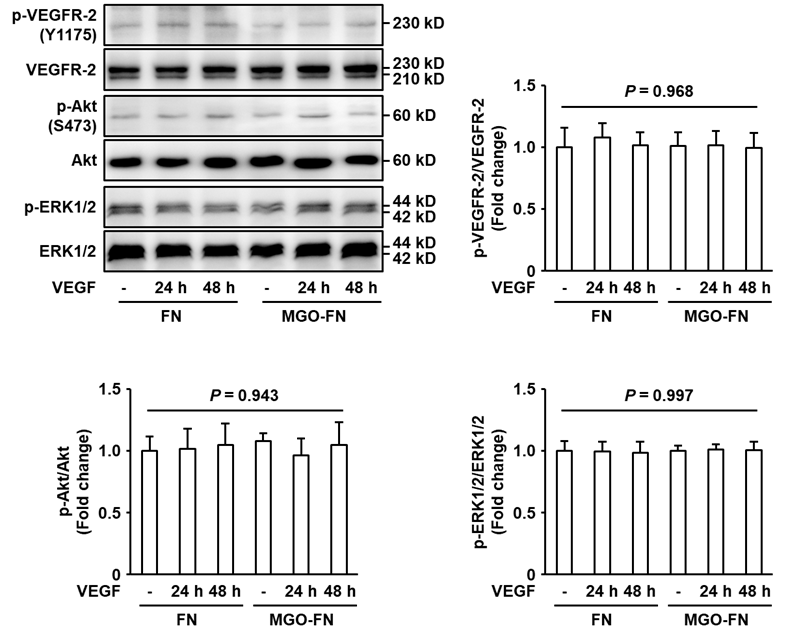
**

**FIGURE S2** The effects of glycated FN on VEGF-induced activation of VEGFR-2 signaling pathway in a long time manner. HUVECs were cultured on FN or MGO-FN and stimulated with VEGF (50 ng/ml) for 24 and 48 h. Phosphorylation (p) of VEGFR-2, Akt and ERK1/2, and total VEGFR-2, Akt and ERK1/2 were analyzed by western blotting in total cell lysates. Representative images of three independent experiments and densitometric analysis of phosphorylated VEGFR-2, Akt and ERK1/2 normalized to total VEGFR-2, Akt and ERK1/2 are shown. All data shown are mean ± SD for triplicate experiments and are expressed as fold changes.

**
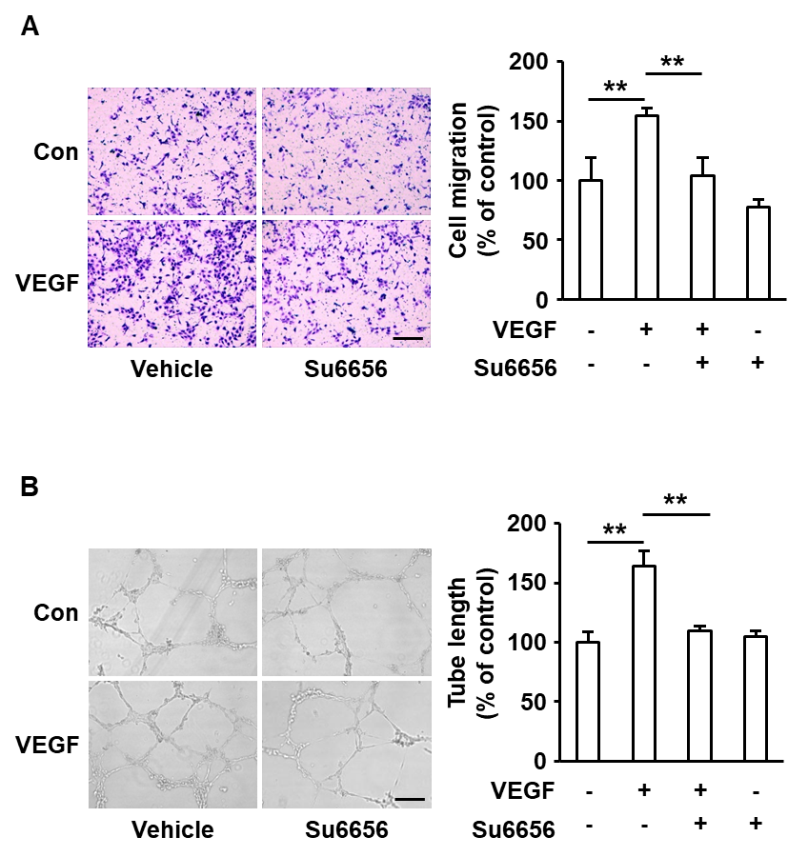
**

**FIGURE S3** Su6656 inhibits VEGF-induced angiogenesis *in vitro*. HUVECs were pretreated with su6656 (5 μM) for 30 min. A, Then cells were added to the upper chambers containing FN-coated porous filters, followed by stimulation with VEGF (50 ng/ml) or vehicle control. After 24 h, the cells were fixed, stained with crystal violet and the cells that had migrated to the lower chambers were counted. Representative images of cell migration are shown. Scale bar is 200 μm. Quantitative assessment of triplicate cell migration experiments was performed. All data shown are mean ± SD and are expressed as % of control. ***P* < .01. B, Cells were seeded onto Matrigel with FN in the presence of VEGF (50 ng/ml) or vehicle control for 24 h. Representative images of tube formation are shown. Scale bar is 500 μm. Quantitative assessment of triplicate tube formation experiments was performed. All data shown are mean ± SD and are expressed as % of control. ***P* < .01.


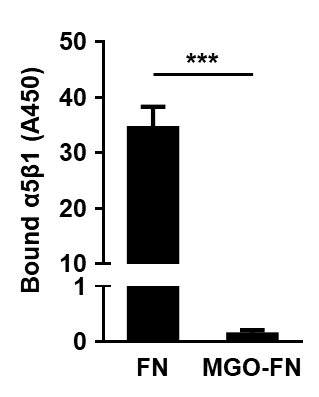


**FIGURE S4** The glycation inhibits the binding between FN and integrin α5β1. 96-well plates were coated with FN (10 μg/mL) or MGO-FN at 4 °C overnight. After washing and blocking, recombinant human integrin α5β1 (5 μg/ml) was added and incubated at 37 °C for 1 h. Bound α5β1 was detected by sequential addition of biotinylated anti-β1 antibody, streptavidin-HRP conjugate and HRP substrate and measurement of OD450. Data shown are mean ± SD for triplicate experiments. ****P* < .001.
